# Mediterranean Diet Adherence and Risk of All-Cause Mortality in Women

**DOI:** 10.1001/jamanetworkopen.2024.14322

**Published:** 2024-05-31

**Authors:** Shafqat Ahmad, M. Vinayaga Moorthy, I-Min Lee, Paul M Ridker, JoAnn E. Manson, Julie E. Buring, Olga V. Demler, Samia Mora

**Affiliations:** 1 Center for Lipid Metabolomics, Brigham and Women’s Hospital, Harvard Medical School, Boston, Massachusetts; 2 Division of Preventive Medicine, Brigham and Women’s Hospital, Harvard Medical School, Boston, Massachusetts; 3 Department of Medical Sciences, Molecular Epidemiology, Uppsala University, Sweden; 4 Department of Epidemiology, Harvard T.H. Chan School of Public Health, Boston, Massachusetts; 5 Department of Computer Science, ETH Zurich, Zürich, Switzerland; 6 Cardiovascular Division, Brigham and Women’s Hospital, Harvard Medical School, Boston, Massachusetts

## Abstract

**Importance:**

Higher adherence to the Mediterranean diet has been associated with reduced risk of all-cause mortality, but data on underlying molecular mechanisms over long follow-up are limited.

**Objectives:**

To investigate Mediterranean diet adherence and risk of all-cause mortality and to examine the relative contribution of cardiometabolic factors to this risk reduction.

**Design, Setting, and Participants:**

This cohort study included initially healthy women from the Women’s Health Study, who had provided blood samples, biomarker measurements, and dietary information. Baseline data included self-reported demographics and a validated food-frequency questionnaire. The data collection period was from April 1993 to January 1996, and data analysis took place from June 2018 to November 2023.

**Exposures:**

Mediterranean diet score (range, 0-9) was computed based on 9 dietary components.

**Main Outcome and Measures:**

Thirty-three blood biomarkers, including traditional and novel lipid, lipoprotein, apolipoprotein, inflammation, insulin resistance, and metabolism measurements, were evaluated at baseline using standard assays and nuclear magnetic resonance spectroscopy. Mortality and cause of death were determined from medical and death records. Cox proportional hazards regression was used to calculate hazard ratios (HRs) for Mediterranean diet adherence and mortality risk, and mediation analyses were used to calculate the mediated effect of different biomarkers in understanding this association.

**Results:**

Among 25 315 participants, the mean (SD) baseline age was 54.6 (7.1) years, with 329 (1.3%) Asian women, 406 (1.6%) Black women, 240 (0.9%) Hispanic women, 24 036 (94.9%) White women, and 95 (0.4%) women with other race and ethnicity; the median (IQR) Mediterranean diet adherence score was 4.0 (3.0-5.0). Over a mean (SD) of 24.7 (4.8) years of follow-up, 3879 deaths occurred. Compared with low Mediterranean diet adherence (score 0-3), adjusted risk reductions were observed for middle (score 4-5) and upper (score 6-9) groups, with HRs of 0.84 (95% CI, 0.78-0.90) and 0.77 (95% CI, 0.70-0.84), respectively (*P* for trend < .001). Further adjusting for lifestyle factors attenuated the risk reductions, but they remained statistically significant (middle adherence group: HR, 0.92 [95% CI, 0.85-0.99]; upper adherence group: HR, 0.89 [95% CI, 0.82-0.98]; *P* for trend = .001). Of the biomarkers examined, small molecule metabolites and inflammatory biomarkers contributed most to the lower mortality risk (explaining 14.8% and 13.0%, respectively, of the association), followed by triglyceride-rich lipoproteins (10.2%), body mass index (10.2%), and insulin resistance (7.4%). Other pathways, including branched-chain amino acids, high-density lipoproteins, low-density lipoproteins, glycemic measures, and hypertension, had smaller contributions (<3%).

**Conclusions and Relevance:**

In this cohort study, higher adherence to the Mediterranean diet was associated with 23% lower risk of all-cause mortality. This inverse association was partially explained by multiple cardiometabolic factors.

## Key Points

**Question:**

Is adherence to the Mediterranean diet associated with lower mortality in a US female population, and if so, what are possible biological mechanisms?

**Findings:**

In this cohort study of 25 315 women followed up for 25 years, higher adherence to the Mediterranean diet was associated with a 23% reduced risk of all-cause mortality. Biomarkers of small molecule metabolites, inflammation, triglyceride-rich lipoproteins, insulin resistance, and body mass index contributed most to explaining this lower risk, with only minimal contributions from standard cholesterol or glycemic measures.

**Meaning:**

In this study, higher adherence to the Mediterranean diet was associated with one-fifth lower relative risk of mortality, which could be partially explained by multiple cardiometabolic risk factors.

## Teaser

This cohort study examines the association between the Mediterranean diet and all-cause mortality over 25 years and evaluates the contribution of different cardiometabolic factors among participants in the Women’s Health Study.

## Introduction

Nutrition and prevention guidelines focus on adherence to dietary patterns rather than single foods in relation to health outcomes.^1,2,3,4,5,6^ A recent umbrella review of 495 unique meta-analyses of observational studies and randomized clinical trials^7^ examined the associations between a wide range of different dietary patterns and cardiometabolic and anthropometric risk factors. The umbrella review evaluated the associations between various diets and cardiometabolic biomarkers. It concluded that among all the diets examined, adherence to the Mediterranean diet demonstrated the most pronounced and consistently beneficial impact on both anthropometric parameters and cardiometabolic risk factors.^7^ The US dietary guidelines have repeatedly designated the Mediterranean diet the healthiest recommended diet.^8^ Guidelines from the American Heart Association, European Society of Cardiology, and Australian National Heart Foundation have consistently highlighted the Mediterranean diet as a healthy dietary pattern for improving cardiometabolic health and cardiovascular disease (CVD) outcomes.^9,10,11,12^

Many large-scale observational epidemiological studies with long follow-up support an association between higher adherence to Mediterranean diet and reduced risk of all-cause mortality.^3,13,14,15,16,17,18,19,20^ A meta-analysis based on 29 observational studies,^21^ with follow-up time ranging from 4 to 32 years and including 1 676 901 participants, reported that a 2-point increase in the consumption of Mediterranean diet was associated with a 10% reduction in all-cause mortality. This standardized approach facilitates comparison across study cohorts, with 1 point assigned for greater-than–study median intake of each of 9 key dietary components of the Mediterranean diet (eg, fruits, vegetables, nuts, and olive oil and monounsaturated fats).

However, long follow-up all-cause mortality data in asymptomatic women are limited. Hence, investigating the association between Mediterranean diet adherence and risk of all-cause mortality in women with extended follow-up is warranted.

Furthermore, the precise mechanisms through which increased adherence to the Mediterranean diet is associated with lower risk of mortality are poorly understood, especially the relative contribution of traditional and newly discovered cardiometabolic biomarkers related to inflammation, lipids and lipoproteins, glucose metabolism and insulin resistance, and branched-chain amino acids (BCAAs). Higher Mediterranean diet adherence was associated with improved inflammatory biomarkers including C-reactive protein (CRP).^22^ The Prevención con Dieta Mediterránea (PREDIMED) randomized clinical trial conducted in Spain, in which participants were followed up for 3 months, reported that Mediterranean diet adherence was associated with reduced oxidized low-density lipoprotein (LDL) cholesterol levels.^23^ A meta-analysis, incorporating data from 19 randomized clinical trials involving 4137 participants and 16 observational studies with 59 001 participants,^24^ found that Mediterranean diet adherence was associated with reduced diastolic and systolic blood pressure. However, the relative contribution of these pathways to lower mortality risk is unknown. Additionally, prior studies did not incorporate new biomarkers representing additional cardiometabolic pathways.

Therefore, in a large-scale epidemiological cohort study of 25 315 initially healthy women from the US population with 25-year follow-up, we aimed to investigate whether higher adherence to Mediterranean diet was associated with lower risk of mortality, and if so, to quantify the contribution of traditional and novel biological biomarkers to the Mediterranean diet–associated mortality reduction. To quantify the mediated effect contributed by both traditional and novel risk factors, we used both standard mediation approaches and counterfactual framework approaches.

## Methods

### Study Population

Our study adhered to the Strengthening the Reporting of Observational Studies in Epidemiology (STROBE) reporting guideline for cohort studies. The Women’s Health Study (WHS) comprised 39 876 female health professionals aged 45 years and older at enrollment (April 30, 1993, to January 24, 1996). Participants were randomly assigned to receive low-dose aspirin, vitamin E, or corresponding placebos to assess the effects on cardiovascular and cancer outcomes (NCT00000479). The trial ended in 2004, with no significant reduction in the primary end points for any treatment, and since then, participants have been followed up on an observational basis, as previously described.^25,26,27^ On the baseline questionnaire, participants provided information about demographic characteristics, anthropometry, lifestyle, medical and social history, and medications. Participants also provided self-reported weight and height, and body mass index (BMI) was calculated as weight in kilograms divided by height in meters squared. Diastolic and systolic blood pressures were reported at baseline. All study participants provided written informed consent, and the study was approved by the institutional review board of Brigham and Women’s Hospital, Boston, Massachusetts.

For the current analysis, we included 28 340 participants who provided blood samples at baseline. We excluded participants who did not have biomarker measurements or dietary information, leaving a total sample of 25 315 women for the current study.

### Mediterranean Diet Adherence

For the assessment of dietary patterns, a validated semiquantitative food-frequency questionnaire of 131 questions was administered to the study participants at baseline.^28^ The Mediterranean diet score has been previously described in detail.^19^ Briefly, the Mediterranean diet score ranged from 0 to 9, with a higher score representing better adherence to Mediterranean diet. This Mediterranean diet score is commonly used for assessing adherence to the Mediterranean diet and is based on regular intake of 9 dietary components. Higher-than-median intake of vegetables (excluding potatoes), fruits, nuts, whole grains, legumes, and fish and the ratio of monounsaturated-to-saturated fatty acids was given 1 point, while the less-than-median intake of red and processed meat was given 1 point. In addition, participants were given 1 point if their intake of alcohol fell within the range of 5 to 15 g/d (otherwise 0 points were assigned). This range approximately corresponds to the consumption of one 5-oz glass of wine, a 12-oz can of regular beer, or 1.5 oz of liquor. Participants were categorized into 3 levels based on Mediterranean diet adherence: scores of 0-3 (low), 4-5 (intermediate), and 6-9 (high),^29^ representing approximate tertiles.

### Mortality Ascertainment

Mortality ascertainment in the WHS cohort has been described in detail.^30,31^ Women completed health questionnaires every 6 months during the first year and annually thereafter. Family members or postal authorities reported most deaths. Medical records and/or death certificates were obtained to confirm causes of these deaths; only deaths with confirmed causes were analyzed for cause-specific analysis. All events were adjudicated according to predefined criteria by an end point committee of physicians. Other deaths were ascertained using the National Death Index. Mortality follow-up is more than 99% complete. CVD mortality included deaths caused by ischemic heart disease, acute myocardial infarction, cerebrovascular disease, sudden death, and other cardiovascular-related deaths.

### Blood Collection and Measurement of Traditional Biomarkers

At baseline, participants’ blood samples were collected in EDTA tubes and shipped overnight to the central laboratory where they were centrifuged and stored at −170 °C until further analyses. The concentrations of hemoglobin A_1c_ in red blood cells were measured through turbidometric assays through Hitachi 911 Analyzer (Roche Diagnostics). The measurement of high-sensitivity CRP (hsCRP) and lipoprotein(a) (Lp[a]) was performed by turbidometric immunoassays through Hitachi 911 analyzer.^26^ Total cholesterol, LDL cholesterol (LDL-C), and high-density lipoprotein cholesterol (HDL-C) were enzymatically measured using assays from Roche Diagnostics and Genzyme. Triglycerides (TG) were measured enzymatically with assays from Roche Diagnostics after correction for endogenous glycerol. Apolipoproteins (apo) AI and B_100_ were measured using turbidometric assays from DiaSorin. Fibrinogen levels were measured with a turbidometric enzymatic assay from R&D Systems. Soluble intracellular adhesion molecule 1 (sICAM-1) levels were enzymatically measured using an immunoassay from R&D Systems. Creatinine levels were measured using a rate-blanked Jaffe reaction-based method from Roche Diagnostics. Homocysteine levels were enzymatically measured using the Hitachi 917 analyzer from Roche Diagnostics with reagents and calibrators from Catch, Inc.

### Nuclear Magnetic Resonance Spectroscopy Metabolomics Biomarkers

We used targeted nuclear magnetic resonance (NMR) spectroscopy^32,33,34^ of ^1^H-NMR (400 MHz) LipoProfile-IV (LipoScience; now LabCorp) to measure lipoprotein subfraction particles of LDL, HDL, very low-density lipoprotein/triglyceride-rich lipoproteins (TRL), and small molecule metabolites. The NMR-based lipoprotein insulin resistance score includes subfractions of TRL, LDL, and HDL particles.^35^ Additionally, the NMR assay was used to measure glycoprotein acetylation (an aggregate inflammatory biomarker of circulating glycosylated acute phase proteins) as well as other cardiometabolic small molecules including alanine, citrate, BCAAs (leucine, isoleucine, valine), and the insulin resistance index, which is a multimarker score that combines lipoprotein insulin resistance and 5-year diabetes risk factor index.

### Statistical Analysis

We used Cox proportional hazards regression models to calculate the adjusted hazards ratio (HRs) and their corresponding 95% CIs with adherence to Mediterranean diet. The low adherence group (score 0-3, approximately bottom tertile) was the reference category. We utilized the median value within each of the 3 Mediterranean diet adherence categories (0-3, 4-5, and 6-9) to evaluate linear trends. A 2-sided *P* > .05 was used for the mediation analysis. The measures of TG, hsCRP, Lp(a), and homocysteine were log-transformed due to nonnormal distribution.

We used the Baron and Kenny approach^36^ to assess whether biomarkers met the criteria for use as mediators. Mediation analyses were conducted using both the standard mediation approach^37^ and counterfactual framework approach^38^ for single biomarkers as mediators to calculate their total mediated effect. However, one of the caveats of using the counterfactual framework approach is that it cannot be applied to multiple biomarkers simultaneously. Person-years of follow-up were calculated from baseline until death or censoring.

We first tested the association of Mediterranean diet adherence with all-cause mortality, with additional analyses for CVD and cancer mortality. Subsequently, we examined the association between adherence to Mediterranean diet and mortality risk using separate models for each potential biomarker. Statistical models were adjusted for age, randomized treatment, and energy intake, with additional adjustments for smoking, physical activity, and menopausal factors in the basic model. The change in magnitude of HRs for the highest (≥6) vs lowest (0-3) Mediterranean diet adherence groups was assessed by adjusting for additional variables one at a time. A greater change in the HR toward the null indicates a larger mediating effect through that particular biomarker on the reduction in mortality risk associated with Mediterranean diet adherence.

Furthermore, on an a priori hypothesis basis, we grouped biomarkers into specific groups based on potential biological mechanisms, as discussed previously.^39^ Then, we evaluated the change in the magnitude of the HRs comparing the lowest with highest Mediterranean diet adherence groups in relation to mortality risk. This was done by subsequently adding 1 group at a time to the basic model, adjusting for baseline age, randomization treatment assignment, energy intake, postmenopausal use of hormones, postmenopausal status, physical activity, and smoking. To assess the mediating effect of each risk factor set on the association between Mediterranean diet adherence and mortality risk, we sequentially added each biomarker group to the basic models. We then compared the change in HRs between the highest and lowest Mediterranean diet adherence groups, both with and without adjustment for each mediator set. An attenuation in the hazard ratios toward null is consistent with an effect mediated by the biomarker (or set of biomarkers) on the association between Mediterranean diet adherence and mortality risk.

The proportion of mortality risk reduction explained by each group of biomarkers was inferred using the formula:

([HR_basic model_ − HR_adjusted model_]/[HR_basic model_ − 1]) × 100%.^37^

Statistical analyses were performed using Stata version 14.0 (StataCorp) and SAS version 9.3 (SAS Institute).

## Results

### Baseline Characteristics

In the current analysis, there were 25 315 female health care professionals with a mean (SD) age of 54.6 (7.1) years at baseline. Self-reported race and ethnicity were as follows: 329 (1.3%) Asian, 406 (1.6%) Black, 240 (0.9%) Hispanic, 24 036 (95.7%) White, and 95 (0.4%) other. The median (IQR) Mediterranean diet score was 4.0 (3.0-5.0). Participants with higher adherence to the Mediterranean diet generally exhibited healthier lifestyles, including lower BMI and higher intake of fruits, nuts, whole grains, legumes, and fish, while consuming less red and processed meat (Table 1). Significant differences were observed in most biomarker and risk factor profiles, with exceptions including systolic blood pressure, LDL-C levels, apo B_100_ levels, LDL particle concentration, and creatinine levels. A higher Mediterranean diet score was associated with an overall healthier biomarker profile.

**Table 1.** Baseline Characteristics and Measures According to Mediterranean Diet Adherence Score

Characteristic	Participants by Mediterranean diet adherence score, median (IQR)			*P* value for trend
	0-3 (n = 9871)	4-5 (n = 9184)	≥6 (n = 6260)	
Age, y	51.9 (48.4-57.3)	53.3 (49.1-59.1)	54.1 (49.8-60.6)	<.001
Current smoking, No. (%)	1547 (15.7)	950 (10.4)	392 (6.3)	<.001
Exercise, No. (%)				
Rarely or never	4411 (44.7)	3251 (35.4)	1626 (26.0)	<.001
<1/wk	2001 (20.3)	1744 (19.0)	1202 (19.2)	
1-3/wk	2662 (27.0)	3096 (33.7)	2410 (38.5)	
≥4/wk	793 (8.0)	1090 (11.9)	1021 (16.3)	
Alcohol consumption, No. (%)				
Rarely	4856 (49.2)	3836 (41.8)	2183 (34.9)	<.001
1-3 drinks/mo	1444 (14.6)	1230 (13.4)	690 (11.0)	
1-6 drinks/wk	2744 (27.8)	3103 (33.8)	2521 (40.3)	
≥1 drinks/d	825 (8.4)	1013 (10.0)	864 (13.8)	
Vegetable intake, servings/d	2.3 (1.6-3.1)	3.7 (2.8-5.0)	5.2 (4.1-6.8)	<.001
Fruits, servings/d	1.3 (0.8-1.8)	2.1 (1.4-2.9)	2.8 (2.2-3.7)	<.001
Nuts, servings/d	0 (0-0.07)	0.07 (0-0.13)	0.07 (0-0.1)	<.001
Whole grains, servings/d	0.7 (0.3-1.1)	1.2 (0.7-1.9)	1.8 (1.3-2.8)	<.001
Legumes, servings/d	0.2 (0.1-0.4)	0.4 (0.2-0.6)	0.6 (0.4-0.9)	<.001
Fish, servings/d	0.1 (0.07-0.2)	0.2 (0.1-0.3)	0.3 (0.2-0.5)	<.001
Ratio of monounsaturated to saturated fat	1.1 (1.0-1.2)	1.1 (1.0-1.2)	1.2 (1.1-1.3)	<.001
Red meat, servings/d	0.6 (0.4-1.0)	0.6 (0.3-1.0)	0.5 (0.3-0.9)	<.001
Processed meats, servings/d	0.1 (0.07-0.3)	0.1 (0-0.2)	0.07 (0-0.2)	<.001
Total calorie intake, kcal	1439.7 (1158.7-1758.4)	1718.7 (1422.1-2063.0)	1990.0 (1677.8-2371.6)	
Postmenopausal status, No. (%)	4909 (49.8)	5064 (55.3)	3685 (59.0)	<.001
BMI	25.0 (22.5-28.3)	24.8 (22.5-28.2)	24.2 (22.1-27.4)	<.001
Blood pressure, mm Hg				
Systolic	125.0 (115.0-135.0)	125.0 (115.0-135.0)	125.0 (115.0-135.0)	.65
Diastolic	80.0 (70.0-80.0)	80.0 (70.0-80.0)	80.0 (70.0-80.0)	.10
Lipids, mg/dL				
LDL cholesterol	121.6 (100.7-144.4)	121.1 (100.9-144.1)	121.4 (100.3-144.7)	.91
HDL cholesterol	51.5 (43.0-61.6)	52.7 (43.9-63.1)	53.8 (44.9-64.5)	<.001
Triglycerides	117.0 (83.0-171.0)	117.0 (83.0-172.0)	116.0 (83.0-167.0)	.15
Total cholesterol	207.0 (183.0-234.0)	208.0 (184.0-235.0)	208.0 (184.0-236.0)	.01
Lipoproteins, mg/dL				
Lipoprotein(a)	10.4 (4.4-32.3)	10.9 (4.7-33.5)	10.8 (4.5-32.9)	.12
Apolipoprotein AI	148.0 (131.3-166.5)	150.0 (133.7-168.5)	152.0 (135.3-171.0)	<.001
Apolipoprotein B_100_	99.8 (83.8-120.4)	99.5 (83.2-120.1)	99.2 (83.5-120.5)	.86
LDL particles and size				
LDL particle concentration, nmol/L	1428.7 (1194.2-1699.1)	1430.0 (1198.8-1695.7)	1433.9 (1194.9-1696.8)	.99
LDL particle size, (nm)	21.0 (20.7-21.3)	21.1 (20.7-21.3)	21.1 (20.8-21.3)	<.001
HDL particles and size				
HDL particle concentration, μmol/L	22.3 (20.2-24.5)	22.5 (20.5-24.7)	22.6 (20.6-24.9)	<.001
HDL particle size, nm	9.0 (8.7-9.3)	9.0 (8.8-9.4)	9.1 (8.8-9.4)	<.001
TRL particles and size				
TRL particle concentration, nmol/L	163.0 (125.2-206.1)	161.3 (122.9-205.6)	160.8 (122.1-206.1)	.13
TRL particle size, nm	42.9 (38.7-48.5)	42.8 (38.8-48.4)	42.7 (38.8-48.0)	.23
Glycemic measures				
Hemoglobin A1c, %	4.99 (4.84-5.18)	4.99 (4.83-5.17)	4.99 (4.82-5.16)	.01
Insulin resistance				
Lipoprotein insulin resistance index score	45.0 (27.0-62.0)	44.0 (26.0-61.0)	42.0 (25.0-59.0)	<.001
5-y diabetes risk factor index score	41.0 (28.0-55.0)	40.0 (28.0-53.0)	39.0 (28.0-52.0)	<.001
Inflammation				
High-sensitivity C-reactive protein, mg/L	2.0 (0.8-4.4)	2.0 (0.8-4.2)	1.8 (0.7-3.9)	<.001
Fibrinogen, mg/dL	350.3 (306.7-403.3)	349.7 (307.5-400.1)	346.1 (304.6-396.7)	.002
Soluble intercellular adhesion molecule 1, ng/mL	344.4 (301.5-397.5)	341.2 (299.8-390.6)	337.1 (297.3-383.5)	<.001
Glycoprotein acetylation, μmol/L	374.7 (330.9-422.4)	372.6 (330.0-418.7)	368.9 (326.1-413.6)	<.001
Branched-chain amino acids, μmol/L				
Total branched-chain amino acids	382.2 (334.6-436.5)	378.6 (333.6-431.7)	376.5 (331.4-427.3)	<.001
Valine	190.1 (165.0-218.4)	188.6 (165.2-215.6)	187.6 (164.6-214.4)	.004
Leucine	128.0 (107.4-150.3)	126.6 (105.6-149.2)	126.2 (106.3-148.3)	.001
Isoleucine	64.7 (53.8-77.3)	64.0 (53.5-76.0)	63.2 (53.2-74.7)	<.001
Small molecule metabolites				
Citrate, μmol/L	99.0 (84.7-114.6)	99.5 (84.3-115.8)	98.2 (83.5-114.8)	.02
Creatinine, mg/dL	0.7 (0.6-0.8)	0.7 (0.6-0.8)	0.7 (0.6-0.8)	.33
Homocysteine, umol/L	10.7 (8.8-13.2)	10.3 (8.6-12.7)	10.2 (8.6-12.4)	<.001
Alanine, mg/dL	3.8 (3.3-4.4)	3.9 (3.4-4.5)	4.0 (3.5-4.5)	<.001

Abbreviations: BMI, body mass index (calculated as weight in kilograms divided by height in meters squared); HDL, high-density lipoprotein; LDL, low-density lipoprotein; TRL, triglyceride-rich lipoprotein.

SI conversion factors: To convert apolipoproteins AI and B_100_ to grams per liter, multiply by 0.01; C-reactive protein to nanomoles per liter, multiply by 9.524; creatinine to micromoles per liter, multiply by 88.4; fibrinogen to micromoles per liter, multiply by 0.0294; HDL, LDL, and total cholesterol to millimoles per liter, multiply by 0.0253; hemoglobin A_1c_ to proportion of total hemoglobin, multiply by 0.01; lipoprotein(a) to micromoles per liter, multiply by 0.0357; and triglycerides to millimoles per liter, multiply by 0.0113.

Most biomarkers met the Baron and Kenny criteria for mediation (Table 1), except for systolic blood pressure, LDL-C level, apo B_100_ level, LDL particle concentration, TRL particle concentration, hemoglobin A_1c_ level, and creatinine. However, given the previously reported associations between higher Mediterranean diet adherence with these biomarkers,^40,41,42^ they were included in the subsequent mediation analyses to assess their association with Mediterranean diet–associated mortality risk (Table 1 and Table 2).

**Table 2.** Association of Mediterranean Diet Adherence With All-Cause Mortality Before and After Adjustment for Risk Factors and Cardiometabolic Biomarkers of Risk

Model	Risk of all-cause mortality by Mediterranean diet score, HR (95% CI)^a^			*P* for trend
	0-3	4-5	≥6	
Age, treatment, and total energy intake–adjusted model	1 [Reference]	0.84 (0.78-0.90)	0.77 (0.70-0.84)	<.001
Age, treatment, and total energy–adjusted model plus each of the following added 1 at a time				
Smoking	1 [Reference]	0.86 (0.80-0.93)	0.81 (0.74-0.93)	<.001
Alcohol consumption	1 [Reference]	0.84 (0.78-0.90)	0.77 (0.70-0.84)	<.001
BMI	1 [Reference]	0.84 (0.78-0.91)	0.78 (0.71-0.85)	<.001
Blood pressure				
Hypertension	1 [Reference]	0.84 (0.78-0.91)	0.77 (0.71-0.84)	<.001
Systolic	1 [Reference]	0.85 (0.79-0.91)	0.78 (0.71-0.85)	<.001
Diastolic	1 [Reference]	0.84 (0.78-0.91)	0.77 (0.71-0.84)	<.001
Traditional lipids				
LDL cholesterol	1 [Reference]	0.84 (0.78-0.90)	0.77 (0.70-0.84)	<.001
HDL cholesterol	1 [Reference]	0.85 (0.78-0.91)	0.78 (0.72-0.85)	<.001
Triglycerides	1 [Reference]	0.84 (0.78-0.90)	0.77 (0.70-0.84)	<.001
Total cholesterol	1 [Reference]	0.84 (0.78-0.90)	0.76 (0.70-0.83)	<.001
Lipoproteins	1 [Reference]			
Lipoprotein(a)	1 [Reference]	0.84 (0.77-0.90)	0.76 (0.70-0.83)	<.001
Apolipoprotein AI	1 [Reference]	0.84 (0.78-0.91)	0.78 (0.71-0.91)	<.001
Apolipoprotein B_100_	1 [Reference]	0.84 (0.78-0.90)	0.77 (0.70-0.84)	<.001
LDL particles and size				
LDL particle concentration	1 [Reference]	0.84 (0.78-0.90)	0.77 (0.70-0.84)	<.001
LDL particle size	1 [Reference]	0.84 (0.78-0.90)	0.76 (0.71-0.84)	<.001
HDL particles and size				
HDL particle concentration	1 [Reference]	0.84 (0.78-0.91)	0.78 (0.71-0.85)	<.001
HDL particle size	1 [Reference]	0.84 (0.78-0.91)	0.78 (0.71-0.85)	<.001
TRL particles and size				
TRL particle concentration	1 [Reference]	0.84 (0.78-0.90)	0.77 (0.70-0.84)	.008
TRL particle size	1 [Reference]	0.84 (0.78-0.90)	0.77 (0.70-0.84)	<.001
Glycemic measures				
Hemoglobin A_1c_	1 [Reference]	0.84 (0.78-0.90)	0.77 (0.71-0.84)	<.001
Insulin resistance				
Lipoprotein insulin resistance index score	1 [Reference]	0.84 (0.78-0.91)	0.78 (0.71-0.85)	<.001
5-y diabetes risk factor index score	1 [Reference]	0.84 (0.78-0.90)	0.76 (0.70-0.83)	<.001
Inflammation				
High-sensitivity C-reactive protein	1 [Reference]	0.84 (0.78-0.90)	0.77 (0.70-0.84)	<.001
Fibrinogen	1 [Reference]	0.84 (0.78-0.91)	0.78 (0.71-0.85)	<.001
Soluble intercellular adhesion molecule 1	1 [Reference]	0.87 (0.80-0.93)	0.81 (0.74-0.89)	<.001
Glycoprotein acetylation	1 [Reference]	0.84 (0.78-0.91)	0.78 (0.71-0.85)	<.001
Branched-chain amino acids				
Total branched-chain amino acids	1 [Reference]	0.84 (0.78-0.90)	0.77 (0.70-0.84)	<.001
Valine	1 [Reference]	0.84 (0.78-0.90)	0.76 (0.70-0.83)	<.001
Leucine	1 [Reference]	0.84 (0.78-0.90)	0.77 (0.70-0.84)	<.001
Isoleucine	1 [Reference]	0.84 (0.78-0.91)	0.77 (0.70-0.84)	<.001
Small molecule metabolites				
Citrate	1 [Reference]	0.84 (0.78-0.90)	0.76 (0.70-0.84)	<.001
Creatinine	1 [Reference]	0.84 (0.78-0.90)	0.76 (0.70-0.84)	<.001
Homocysteine	1 [Reference]	0.85 (0.79-0.92)	0.78 (0.72-0.86)	<.001
Alanine	1 [Reference]	0.84 (0.78-0.91)	0.77 (0.71-0.84)	<.001

Abbreviations: BMI, body mass index (calculated as weight in kilograms divided by height in meters squared); HDL, high-density lipoprotein; HR, hazard ratio; LDL, low-density lipoprotein; TRL, triglyceride-rich lipoprotein.

^a^ Participants were categorized according to 3 levels of Mediterranean diet adherence score (scores of 0-3, 4-5, and 6-9). *P* values across 3 levels of Mediterranean diet adherence were all less than .05.

### Mediterranean Diet Adherence and Lower Risk of Mortality

**Figure 1.**
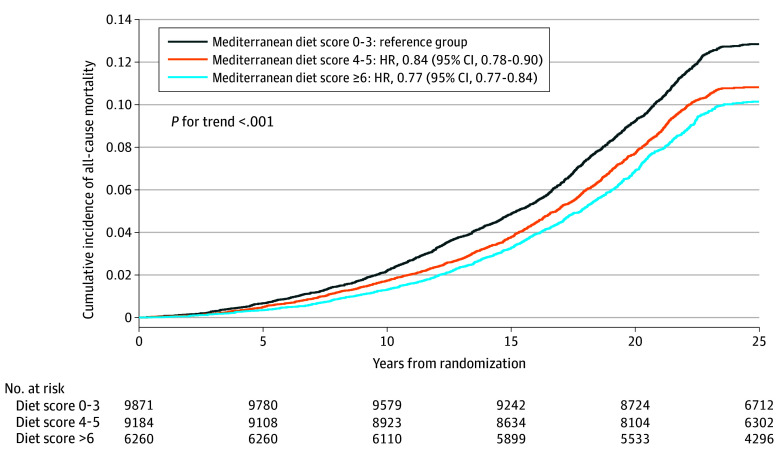
Cumulative Survival for the Mediterranean Diet in All-Cause Mortality–Confirmed Person Years The analyses were adjusted for age, treatment, and total energy intake.

During a mean (SD) follow-up of 24.7 (4.8) years, a total of 3879 all-cause deaths occurred, including 935 CVD deaths and 1531 cancer deaths. A higher Mediterranean diet score was associated with decreased risks of all-cause, CVD, and cancer mortality in a linear trend (eTable 1 in Supplement 1). Cumulative incidence curves for the Mediterranean diet score with each of all-cause, CVD, and cancer mortality risks are shown in Figure 1 and eFigure 1 in Supplement 1. Compared with women whose scores were 3 or less, higher adherence to the Mediterranean diet was associated with reduced all-cause mortality, with HRs of 0.84 (95% CI, 0.78-0.90) for women with scores of 4 to 5 and 0.77 (95% CI, 0.70-0.84) for scores of 6 or greater (*P* for trend < .001) (Table 3). A stronger association for cancer mortality and adherence to the Mediterranean diet was observed than for CVD mortality. Specifically, higher scores (≥6) were associated with reduced risk of CVD mortality (HR, 0.83 [95% CI, 0.69-0.99]) (*P* for trend = .03) and cancer mortality 0.80 (95% CI, 0.69-0.92) (*P *for trend = .002) (eTable 1 in Supplement 1).

**Table 3.** Association of Mediterranean Diet Adherence With All-Cause Mortality Events Before and After Adjustment for Sets of Potential Mediators

Model	Risk of all-cause mortality by Mediterranean diet adherence score, HR (95% CI)			*P* for trend
	Score 0-3	Score 4-5	Score ≥6	
Age, treatment, and total energy intake–adjusted model	1 [Reference]	0.84 (0.78-0.90)	0.77 (0.70-0.84)	<.001
Basic model^a^	1 [Reference]	0.92 (0.85-0.99)	0.89 (0.82-0.98)	.001
Basic model plus each set of risk factors below, added one group at a time^b^				
BMI	1 [Reference]	0.92 (0.85-1.00)	0.90 (0.82-0.99)	.02
Hypertension	1 [Reference]	0.92 (0.86-1.00)	0.90 (0.82-0.98)	.001
Apolipoproteins: lipoprotein(a), apolipoprotein AI, apolipoprotein B_100_	1 [Reference]	0.92 (0.85-0.99)	0.89 (0.81-0.98)	.01
LDL measures: LDL particle size and concentration, LDL cholesterol, apolipoprotein B_100_	1 [Reference]	0.92 (0.85-0.99)	0.90 (0.82-0.98)	.01
HDL measure: HDL particle size and concentration, HDL cholesterol, apolipoprotein AI	1 [Reference]	0.92 (0.85-0.99)	0.90 (0.82-0.98)	.01
TRL measures: TRL particle size and concentrations, triglycerides	1 [Reference]	0.92 (0.86-1.00)	0.90 (0.82-0.99)	.02
Hemoglobin A_1c_	1 [Reference]	0.92 (0.85-0.99)	0.89 (0.82-0.98)	.01
Insulin resistance: lipoprotein insulin resistance index score, 5-y diabetes risk factor index score	1 [Reference]	0.92 (0.86-1.00)	0.90 (0.82-0.99)	.02
Inflammation: hsCRP, fibrinogen, sICAM-1, glycoprotein acetylation	1 [Reference]	0.93 (0.86-1.00)	0.91 (0.83-0.99)	.03
Branched-chain amino acids	1 [Reference]	0.92 (0.85-0.99)	0.90 (0.82-0.98)	.01
Small-molecule metabolites: citrate, creatinine, homocysteine, alaline	1 [Reference]	0.93 (0.86-1.00)	0.91 (0.83-1.00)	.03
All of the above^c^	1 [Reference]	0.93 (86.0-1.01)	0.92 (0.83-1.00)	.048

Abbreviations: BMI, body mass index; HDL, high-density lipoprotein; HR, hazard ratio; hsCRP, high-sensitivity C-reactive protein; LDL, low-density lipoprotein; sICAM-1, soluble intracellular adhesion molecule 1; TRL, triglyceride-rich lipoprotein.

^a^ Basic model included age, randomized treatment assignment; total energy intake (quintiles), smoking, menopausal status, postmenopausal hormone use, and physical activity. Participants were followed up for up to 25 years from baseline.

^b^ Models were adjusted for the variables in the basic model plus each of the sets of risk factors added one group at a time to separate models.

^c^ Model included variables in the basic model, plus all sets of risk factors included simultaneously in 1 model.

After additional adjustment for potential confounders (smoking, physical activity, alcohol intake, and menopausal factors), HRs remained significant for all-cause mortality, with values of 0.92 (95% CI, 0.85-0.99) and 0.89 (95% CI, 0.82-0.98) for women with scores of 4 to 5 and 6 or greater, respectively, compared with those with 3 or less (*P* for trend = .001) (eTable 1 in Supplement 1). Associations for cancer and CVD mortality were generally attenuated. Similar results were observed for per-unit increment in the score with mortality (eTable 2 in Supplement 1).

**Figure 2.**
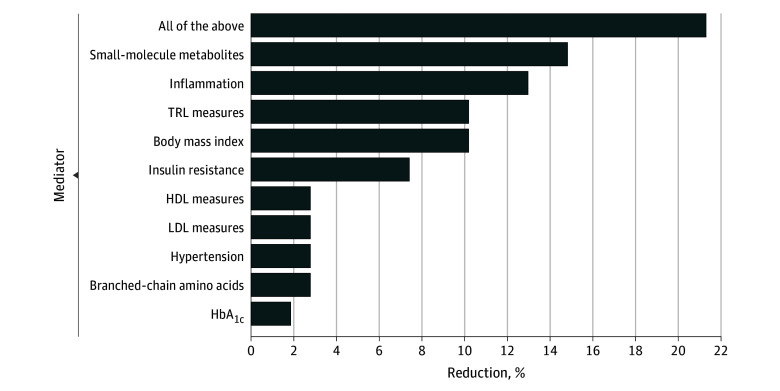
The Proportion of All-Cause Mortality Reduction for High Adherence to the Mediterranean Diet Attributed to Groups of Mediators Mediator groups were small-molecule metabolites (citrate, creatinine, homocysteine, alaline), inflammation (high-sensitivity C-reactive protein, fibrinogen, soluble intracellular adhesion molecule 1, and glycoprotein acetylation), triglyceride-rich lipoproteins (TRL) measures (triglyceride-rich lipoprotein particle size and concentrations, triglycerides), body mass index (calculated as weight in kilograms divided by height in meters squared), insulin resistance (lipoprotein insulin resistance index score, 5-y diabetes risk factor index score), high-density lipoprotein (HDL) measures (HDL particle size and concentration, HDL cholesterol), low-density lipoprotein (LDL) measures (LDL particle size and concentration, LDL cholesterol), hypertension, branched-chain amino acids, and hemoglobin A_1c_ (HbA_1c_). Apolipoproteins (lipoprotein[a], apolipoprotein AI, and apolipoprotein B_100_) did not contribute to mediating the association of Mediterranean diet adherence with all-cause mortality. The basic model included age, randomized treatment assignment, energy intake, smoking, alcohol intake, menopausal status, postmenopausal hormone use, and physical activity.

In the follow-up analyses, Cox regression models were adjusted individually for each risk factor or biomarker one at a time in addition to age, treatment, and energy intake (Table 3). While some results were no longer statistically significant, most remained significant for all-cause mortality. Next, to better characterize the extent to which the reduced risk of all-cause mortality associated with Mediterranean diet adherence was affected by potential sets of mediators from different physiological pathways, we grouped the risk factors and biomarkers into physiological sets, and each set of mediators was added one at a time to the basic model (Table 3 and Figure 2). We computed the degree to which the association of the adherence score with all-cause mortality could be attributed to each set of potential mediators. For all-cause mortality, we observed that small-molecule metabolites (in particular homocysteine and alanine) and inflammation explained the largest contributions to the lower risk of mortality associated with the Mediterranean diet (14.8% and 13.0%, respectively), with lesser contributions from TRLs (10.2%), BMI (10.2%), and insulin resistance (7.4%). Smaller contributions (<3%) were seen for HDL or LDL measures, hypertension, BCAAs, and hemoglobin A_1c_ levels. When all the risk factors and biomarkers were added together in the regression model, we observed a 21.3% total mediation effect.

In sensitivity analyses, we compared the reported mediation results for single biomarkers through both the counterfactual framework approach^38^ as well as the standard mediation approach. The results were generally similar across both approaches (eTable 3 and eFigure 2 in Supplement 1).

## Discussion

In this large-scale cohort study of 25 315 initially healthy US women who were followed up for 25 years, we observed that higher adherence to the Mediterranean diet was associated with a 23% relative risk reduction in all-cause mortality. Furthermore, this risk reduction was explained partially by potential mediation through small molecules metabolites (eg, alanine), inflammatory biomarkers, TRL measures, insulin resistance, and BMI and, to a much lesser extent, by blood pressure, HDL, LDL, apo B_100_, Lp(a), or glycemic measures.

Our findings of lower risk of all-cause mortality among women with higher adherence to the Mediterranean diet are consistent with the data from prior studies in US populations, which reported that higher Mediterranean diet consumption was associated with 16% reductions in all-cause and CVD mortality,^14^ and other cohorts based in the US and non-US populations have reported beneficial effects of the Mediterranean diet.^13,19,20,43^ Another meta-analysis of 21 cohort studies,^44^ which included 883 878 participants, reported that higher Mediterranean diet adherence was associated with 21% reduced risk of CVD mortality. The study findings with long follow-up mortality in this population of women are also consistent with our prior study evaluating risk of CVD events, which found one-quarter reduction in total CVD events (fatal and nonfatal) over a 12-year period for the top vs bottom Mediterranean diet adherence groups.^32^ However, we observed that the association of Mediterranean diet adherence with cancer mortality was generally stronger than that with CVD mortality. Similar to our findings, the UK Biobank study found that Mediterranean diet adherence was associated with lower cancer mortality than CVD mortality.^45^

Prior shorter-term studies have also demonstrated beneficial effects of Mediterranean diet adherence in relation to cardiometabolic, inflammatory, and lipid biomarkers. In a cross-sectional study, adherence to the Mediterranean diet was associated with lower levels of CRP and interleukin 6 (IL-6) and improved endothelial function.^22,46,47^ A 2-year Mediterranean diet intervention found significant lowering of CRP, IL-6, IL-7, and IL-18 as well as improved insulin resistance.^48^ These prior studies are consistent with the current findings of the mediation of associations for inflammatory and insulin-resistance biomarkers. In most prior studies, Mediterranean diet adherence did not result in substantial changes in total cholesterol, LDL cholesterol, or Lp(a), consistent with the current results. In a 3-month dietary clinical trial,^23^ Mediterranean diet consumption was better in reducing oxidized LDL levels in comparison with a low-fat diet. It is possible that more functional assessments of LDL and HDL may be related to the Mediterranean diet benefit.

The current study was a large-scale cohort epidemiological study with validated dietary measures, detailed and comprehensive measures of traditional and novel NMR-based biomarkers, and a large number of deaths (including CVD and cancer deaths) during 25 years of follow-up of US women. For mediation analysis aimed at understanding the contribution of individual or sets of biomarkers to the association of Mediterranean diet adherence with all-cause mortality, we utilized both traditional and counterfactual framework approaches, with similar results of the 2 methods. Since we utilized the National Death Index as well as medical record review and next-of-kin reports, the lost to mortality follow-up is negligible (less than 1%).

### Limitations

This study has several potential limitations. The study participants were middle aged and older well-educated female health professionals who were predominantly non-Hispanic White individuals, which may limit the generalizability of the findings. Dietary adherence was assessed through food-frequency questionnaires, and we cannot rule out the possibility of exposure misclassification. Dietary assessments and blood biomarker assays were conducted at baseline as follow-up blood samples were not collected. Potential residual confounding from unmeasured variables cannot be ruled out. Furthermore, it is plausible that certain covariates, such as hypertension and BMI, could act as confounders and/or potential mediators. While adherence to the Mediterranean diet did not specifically include certain dietary components such as trans-fat, glycemic load, and polyunsaturated fatty acids, these components are likely to be correlated with the foods incorporated into the score. Additionally, the anthropometric measures, including height, weight, and blood pressure, were self-reported, although the questionnaires used have been validated previously in female health care professionals.^49,50^

## Conclusions

In summary, in this large-scale study of initially healthy US women there was 23% lower relative risk of all-cause mortality comparing women with scores of 6 or greater and with scores of 3 or less for Mediterranean diet adherence. Our results suggest that a proportion of the lower risk of mortality may be accounted for by several cardiometabolic risk factors, in particular, biomarkers related to metabolism, inflammation, TRL pathways, insulin resistance, and BMI, but not those related to total cholesterol, LDL-C, Lp(a), or standard glycemic measures, such as hemoglobin A_1c_. Despite this, most of the potential benefit of adherence to the Mediterranean diet and morality remains unexplained, and future studies should examine other pathways that could potentially mediate the Mediterranean diet–associated lower mortality as well as examine cause-specific mortality.

## Notes

Supplement 1. **eTable 1.** Association of Mediterranean Diet Adherence Score With All-Cause and CVD Mortality Events After Adjustment for Sets of Potential Mediators (Total Years Follow-Up) **eTable 2.** Association of Per-Unit Increment in Mediterranean Diet Adherence With All-Cause and CVD Mortality Events After Adjustment for Sets of Potential Mediators (Total Years Follow-Up) **eTable 3.** Mediation Effect Explained Through Different Risk Factors Regarding Mediterranean Diet Adherence With All-Cause Mortality Events **eFigure 1.** Cumulative Survival for the Mediterranean Diet Adherence in CVD and Cancer Mortality Confirmed Person-Years **eFigure 2.** Mediation Effect Explained for Different Risk Factors Using Counterfactual Framework Approach and Standard Mediation Approach Regarding Mediterranean Diet Adherence With All-Cause Mortality Events

Supplement 2.
Data Sharing Statement
