# Supplementary material for: Mediterranean Diet Adherence and Risk of All-Cause Mortality in Women
Source: JAMA Netw Open. 2024 May 31;7(5):e2414322. doi: 10.1001/jamanetworkopen.2024.14322 (PMC11143458; doi:10.1001/jamanetworkopen.2024.14322)
Supplement: Supplement 1. — eTable 1. Association of Mediterranean Diet Adherence Score With All-Cause and CVD Mortality Events After Adjustment for Sets of Potential Mediators (Total Years Follow-Up) eTable 2. Association of Per-Unit Increment in Mediterranean Diet Adherence With All-Cause and CVD Mortality Events After Adjustment for Sets of Potential Mediators (Total Years Follow-Up) eTable 3. Mediation Effect Explained Through Different Risk Factors Regarding Mediterranean Diet Adherence With All-Cause Mortality Events eFigure 1. Cumulative Survival for the Mediterranean Diet Adherence in CVD and Cancer Mortality Confirmed Person-Years eFigure 2. Mediation Effect Explained for Different Risk Factors Using Counterfactual Framework Approach and Standard Mediation Approach Regarding Mediterranean Diet Adherence With All-Cause Mortality Events [file jamanetwopen-e2414322-s001.pdf]

Supplemental Online Content

Ahmad S, Moorthy MV, Lee I, et al. Mediterranean diet adherence and risk of all-cause mortality in women. *JAMA Netw Open.* 2024;7(5):e2414322. doi:10.1001/jamanetworkopen.2024.14322

eTable 1. Association of Mediterranean Diet Adherence Score With All-Cause and CVD Mortality Events After Adjustment for Sets of Potential Mediators (Total Years Follow-Up)

eTable 2. Association of Per-Unit Increment in Mediterranean Diet Adherence With All-Cause and CVD Mortality Events After Adjustment for Sets of Potential Mediators (Total Years Follow-Up)

eTable 3. Mediation Effect Explained Through Different Risk Factors Regarding Mediterranean Diet Adherence With All-Cause Mortality Events

eFigure 1. Cumulative Survival for the Mediterranean Diet Adherence in CVD and Cancer Mortality Confirmed Person-Years

eFigure 2. Mediation Effect Explained for Different Risk Factors Using Counterfactual Framework Approach and Standard Mediation Approach Regarding Mediterranean Diet Adherence With All-Cause Mortality Events

This supplemental material has been provided by the authors to give readers additional information about their work.

© 2024 Ahmad S et al. *JAMA Network Open.*

eTable 1. Association of Mediterranean Diet Adherence Score With All-Cause and CVD Mortality Events After Adjustment for Sets of Potential Mediators (Total Years Follow-Up)

**All-cause Mortality**				
	Score 0-3	Score 4-5	Score ≥ 6	P for Trend
Age, treatment, and total energy intake- adjusted model	1 [Reference]	0.84 (0.78-0.90)	0.77 (0.70-0.84)	<0.001
Basic model*	1 [Reference]	0.92 (0.85-0.99)	0.89 (0.82-0.98)	0.001
**Cancer Mortality**				
	Score 0-3	Score 4-5	Score ≥ 6	P for Trend
Age, treatment, and total energy intake- adjusted model	1 [Reference]	0.90 (0.80-1.02)	0.80 (0.69-0.92)	0.002
Basic model*	1 [Reference]	0.98 (0.87-1.10)	0.92 (0.79-1.06)	0.25
**CVD Mortality**				
	Score 0-3	Score 4-5	Score ≥ 6	P for Trend
Age, treatment, and total energy intake- adjusted model	1 [Reference]	0.86 (0.74-1.01)	0.83 (0.69-0.99)	0.033
Basic model*	1 [Reference]	0.95 (0.81-1.11)	0.96 (0.80-1.16)	0.65

*Basic Model included age, randomized treat assignment; total energy intake (TEI, quintiles), smoking, menopausal status, postmenopausal hormone use, alcohol use, and physical activity. Participants were followed-up upto 25 years from baseline.

© 2024 Ahmad S et al. *JAMA Network Open.*

eTable 2. Association of Per-Unit Increment in Mediterranean Diet Adherence With All-Cause and CVD Mortality Events After Adjustment for Sets of Potential Mediators (Total Years Follow-Up)

All-cause mortality

	HR (95%CIs)	P for Trend
Age, treatment, and total energy intake- adjusted model	0.94 (0.92-0.95)	<0.001
Basic model*	0.97 (0.95-0.99)	0.002

Cancer-mortality

	HR (95%CIs)	P for Trend
Age, treatment, and total energy intake- adjusted model	0.95 (0.92-0.98)	0.001
Basic model*	0.98 (0.95-1.01)	0.22

CVD-mortality

	HR (95%CIs)	P for Trend
Age, treatment, and total energy intake- adjusted model	0.95 (0.92-0.99)	0.015
Basic model*	0.99 (0.94-1.02)	0.33

*Basic Model included age, randomized treat assignment; total energy intake (TEI, quintiles), smoking, menopausal status, postmenopausal hormone use, alcohol use, and physical activity. Participants were followed-up upto 25 years from baseline.

© 2024 Ahmad S et al. *JAMA Network Open.*

eTable 3. Mediation Effect Explained Through Different Risk Factors Regarding Mediterranean Diet Adherence With All-Cause Mortality Events

Basic Model	**% Mediation effect explained through Counterfactual Framework**	**% Mediation effect explained through standard approach**
BMI (kg/m^2)	6.3	10.0
**Blood Pressure**		
Systolic blood pressure, mmHg	8.2	6.7
Diastolic blood pressure, mmHg	5.0	3.3
**Lipids**		
LDL cholesterol, mg/dL	0.12	0.0
HDL cholesterol, mg/dL	3.2	3.3
Triglycerides, mg/dL	0.9	3.3
Total cholesterol, mg/dL	0.2	0.0
**Apolipoprotein**		
Lipoprotein(a), mg/dL	-0.7	0.0
Apolipoprotein A1, mg/dL	1.8	0.0
Apolipoprotein B-100, mg/dL	-0.04	0.0
**LDL particles and size**		
cLDLP, nmol/L	0.0	0.0
LDLZ, nm	1.8	3.3
**HDL particles and size**		
cHDLP, µmol/L	0.9	0.0
HDLZ, nm	1.1	3.3
**TRL particles and size**		
TRLP, nmol/L	-0.7	0.0
TRLZ, nm	5.5	10.0
**Glycemic**		
Hemoglobin A1c	3.2	3.3
**Insulin resistance**		
LPIR, Score 1-100	4.3	6.7
DRI, Score 1–100	2.6	6.7
**Inflammation pathway**		
High-sensitivity CRP, mg/L	6.0	10.0
Fibrinogen, mg/dL	0.7	0.0
Soluble ICAM-1, ng/mL	9.6	13.3
**Branched-chain amino acids**		
Total BCAA, µmol/L	0.3	0.0
Valine, µmol/L	3.2	0.0
Leucine, µmol/L	0.3	0.0
Isoleucine, µmol/L	2.2	3.3
**Small molecule metabolites**		
Citrate, µmol/L	-0.3	0.0
Creatinine, mg/dL	-0.1	0.0
Homocysteine, umol/L	6.0	10.0
Alanine, mg/dL	5.5	6.7

*Basic Model included age, randomized treat assignment; total energy intake (TEI, quintiles), smoking, menopausal status, postmenopausal hormone use, alcohol use, physical activity.

© 2024 Ahmad S et al. *JAMA Network Open.*

Abbreviations: BMI, body mass index (calculated as weight in kilograms divided by height in 1 meters squared); HDL, high-density lipoprotein; HR, hazard ratio; LDL, low-density 2 lipoprotein; TRL, triglyceride-rich lipoprotein; LPIR, lipoprotein insulin resistence; DRI, 5-years diabetes risk factor index; CRP, C-reactive protein; ICAM-1, Soluble intracellular adhesion molecule 1; BCAA, branched-chain amino acids.

© 2024 Ahmad S et al. *JAMA Network Open.*

eFigure 1. Cumulative Survival for the Mediterranean Diet Adherence in CVD and Cancer Mortality Confirmed Person-Years

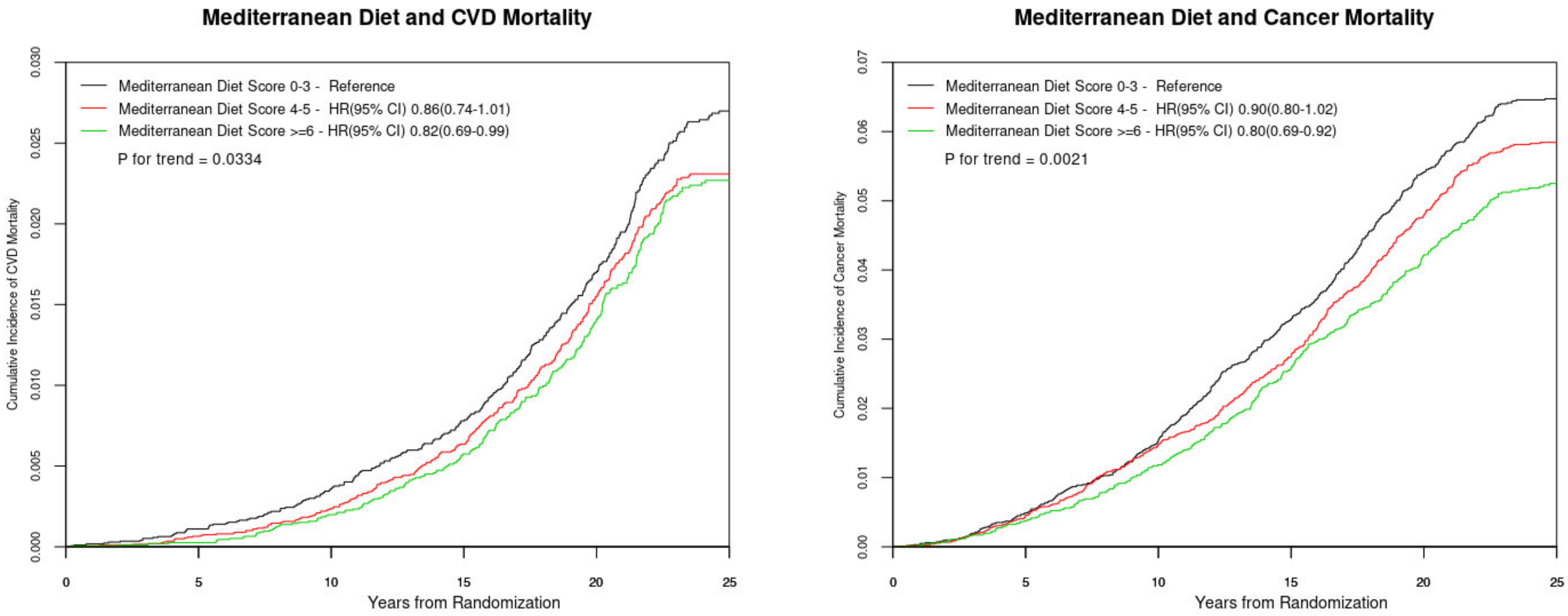

The analyses were adjusted for age, treatment, and total energy intake.

© 2024 Ahmad S et al. *JAMA Network Open.*

eFigure 2. Mediation Effect Explained for Different Risk Factors Using Counterfactual Framework Approach and Standard Mediation Approach Regarding Mediterranean Diet Adherence With All-Cause Mortality Events

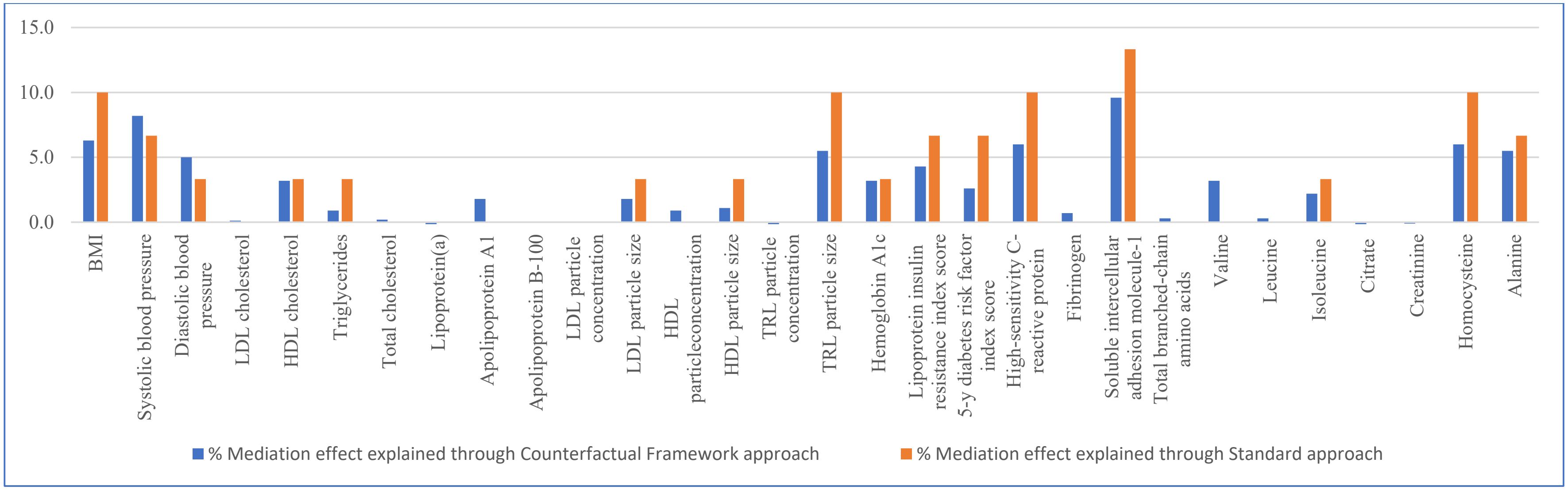

Abbreviations: BMI, body mass index (calculated as weight in kilograms divided by height in 1 meters squared); HDL, high-density lipoprotein; HR, hazard ratio; LDL, low-density 2 lipoprotein; TRL, triglyceride-rich lipoprotein.

© 2024 Ahmad S et al. *JAMA Network Open.*
