# Supplementary material for: Mediterranean Diet Adherence and Risk of All-Cause Mortality in Women
Source: JAMA Netw Open. 2024 May 31;7(5):e2414322. doi: 10.1001/jamanetworkopen.2024.14322 (PMC11143458; doi:10.1001/jamanetworkopen.2024.14322)
Supplement: Supplement 2. — Data Sharing Statement [file jamanetwopen-e2414322-s002.pdf]

Data Sharing Statement

Ahmad. Mediterranean Diet Adherence and Risk of All-Cause Mortality in Women. *JAMA Netw Open*. Published May 31, 2024. doi:10.1001/jamanetworkopen.2024.14322

Data
Data available: No

Additional Information
Explanation for why data not available: Deidentified limited dataset requests upon review and approval by the Women's Health Study Publications Committee.
